# Heat shock factor HSFB2a involved in gametophyte development of *Arabidopsis thaliana* and its expression is controlled by a heat-inducible long non-coding antisense RNA

**DOI:** 10.1007/s11103-014-0202-0

**Published:** 2014-05-30

**Authors:** Markus Wunderlich, Rita Groß-Hardt, Friedrich Schöffl

**Affiliations:** 1 ZMBP General Genetics, University of Tübingen, 72076 Tübingen, Germany; 2 ZMBP Developmental Genetics, University of Tübingen, 72076 Tübingen, Germany

**Keywords:** HSFB2a, Heat shock factor, Gametophyte development, Antisense RNA, *Arabidopsis thaliana*

## Abstract

Heat stress transcription factors (HSFs) are central regulators of the heat stress response. Plant HSFs of subgroup B lack a conserved sequence motif present in the transcriptional activation domain of class A-HSFs. Arabidopsis members were found to be involved in non-heat shock functions. In the present analysis we investigated the expression, regulation and function of *HSFB2a. HSFB2a* expression was counteracted by a natural long non-coding antisense RNA, *asHSFB2a*. In leaves, the antisense RNA gene is only expressed after heat stress and dependent on the activity of HSFA1a/HSFA1b. *HSFB2a* and *asHSFB2a* RNAs were also present in the absence of heat stress in the female gametophyte. Transgenic overexpression of *HSFB2a* resulted in a complete knock down of the *asHSFB2a* expression. Conversely, *asHSFB2a* overexpression leads to the absence of *HSFB2a* RNA. The knockdown of *HSFB2a* by *asHSFB2a* correlated with an improved, knockdown of *asHSFB2a* by *HSFB2a* overexpression with an impaired biomass production early in vegetative development. In both cases the development of female gametophytes was impaired. A T-DNA knock-out line did not segregate homozygous mutant plants, only heterozygots *hsfB2a*-*tt1*/+ were viable. Approximately 50 % of the female gametophytes were arrested in early development, before mitosis 3, resulting in 45 % of sterile ovules. Our analysis indicates that the “Yin–Yang” regulation of gene expression at the *HSFB2a* locus influences vegetative and gametophytic development in Arabidopsis.

**Electronic supplementary material:**

The online version of this article (doi:10.1007/s11103-014-0202-0) contains supplementary material, which is available to authorized users.

## Introduction

Heat stress transcription factors (HSFs) were originally identified as the central regulators of the heat stress response. In recent years HSFs have been found to be involved in the control of a multitude of stress responses and also in developmental processes. Unlike in other eukaryotes, there is a high diversity of HSFs in plants with 21 genes in *Arabidopsis* (Nover et al. 2001), 25 in rice (Guo et al. 2008), 30 in maize (Lin et al. 2011; Scharf et al. 2012) and 52 in soybean (Scharf et al. 2012) respectively. This may reflect a functional redundancy in controlling stress responses, evident for the A1-group of HSFs in *Arabidopsis* (Liu et al. 2011; Yoshida et al. 2011) and specific functions in non-heat stress processes as demonstrated for maize (Gagliardi et al. 1995) rice (Chauhan et al. 2011) and Arabidopsis (Kotak et al. 2007).

Common features of all HSFs are the highly conserved DNA binding domain (DBD) that mediates binding to the heat stress elements (HSEs), repetitions of the “nGAAnnTTCn” motif in promoters of target genes (Schöffl et al. 1998) and two hydrophobic regions A and B, that in case of plant class B HSFs are separated by a short linker (Nover et al. 2001). Plant HSFs are grouped in three classes A, B and C. Whereas almost all class A HSFs hold one or two acidic AHA motifs conferring competence as transcriptional activators (Döring et al. 2000), all class B HSFs lack this domain and instead have a B3 repressing domain that was also found in 24 other transcription factors of *Arabidopsis* (Czarnecka-Verner et al. 2004; Ikeda and Ohme-Takagi 2009).

The importance of HSF in developmental regulation in *Arabidopsis* has been demonstrated for HSFB4, which acts mainly in the root stem cell niche to control cell identity and cell fate (Begum et al. 2012; Pernas et al. 2010; ten Hove et al. 2010). On the other hand, *Arabidopsis* HSFB2b, together with HSFB1, was shown to be involved in the regulation of the defensin gene *PDF1.2* and pathogen resistance (Kumar et al. 2009).

These HSFs are also negative regulators of HSFA2, HSFA7a, and act in an autoregulatory manner (Ikeda et al. 2011). Only these class B HSFs have also been found to elicit mild cell death effects in *N. benthamiana* leaves (Zhu et al. 2012). For HSFB2a a mild repressing activity was also shown at the *HSFA2* promoter (Ikeda et al. 2011), which is expressed in antipodal cells of mature female gametophytes (Kägi et al. 2010).

In this study we provide evidence that HSFB2a is required for the development of the female germ line and that its expression is controlled by a natural heat-inducible non-coding antisense RNA and vice versa.

## Results

### *HSFB2a* is required for plant fertility

**Fig. 1:**
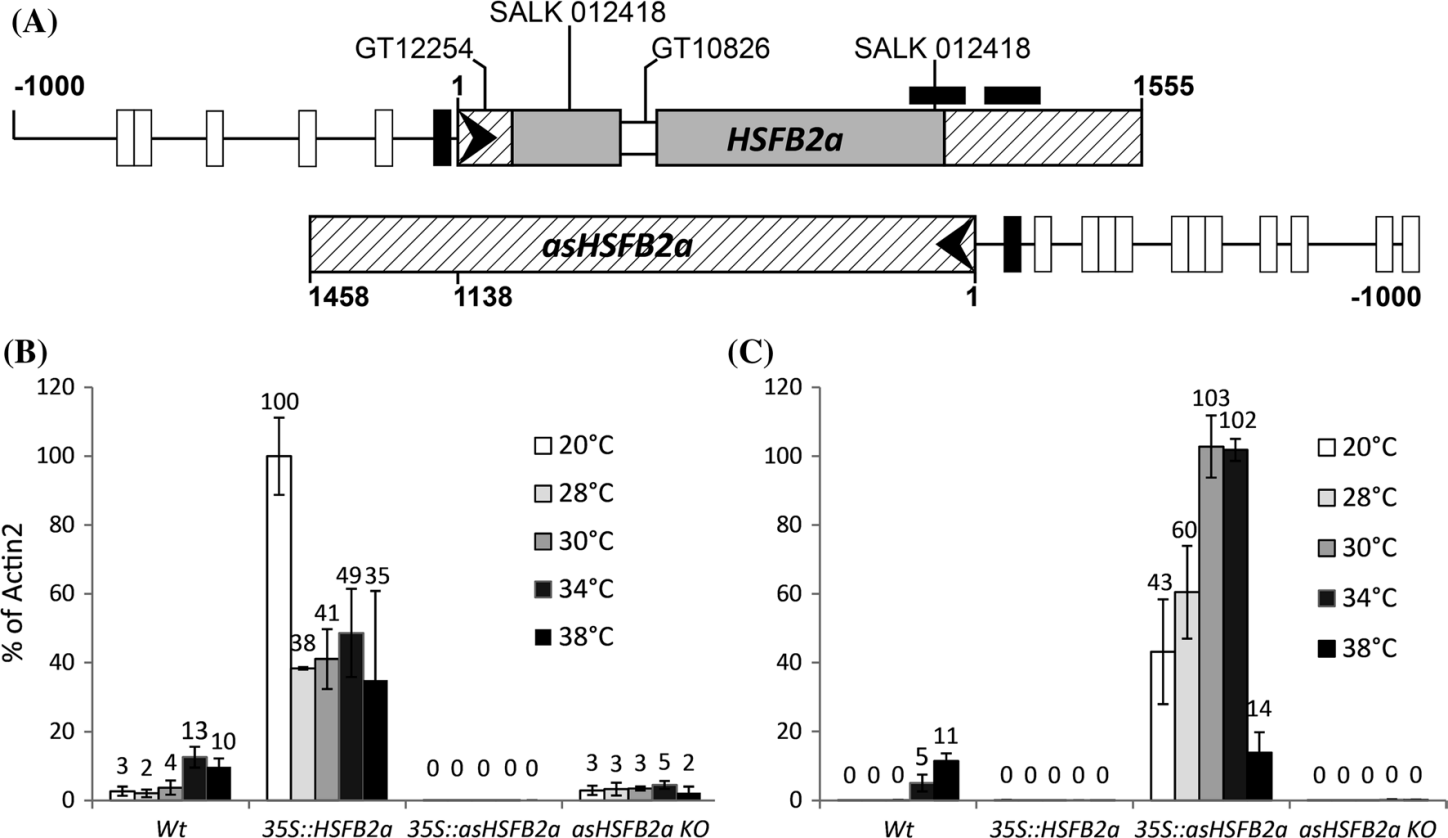
**A** Position of the T-DNA insertions in the *HSFB2a* genomic region. *Grey boxes* depict the coding sequence, the *open box* the intron, hatched boxes the 5′-UTR and 3′-UTR, and *arrowheads* the direction of transcription. *Black upright boxes* mark perfect, *open boxes* imperfect HSEs. *Crosswise black boxes* show the position of in situ hybridization probes. *Arrows* denote primer positions, numbers refer to nucleotide positions relative to the transcription start and lines indicate the insertion site of the respective T-DNA. The expression levels of *HSFB2a* (**B**) and *asHSFB2a* (**C**) mRNA in wild-type and the respective overexpressing and knockout (SALK_012418) plants at different temperatures are indicated by the *numbers* above the columns. All values were normalized with respect to *Actin2* mRNA (=100 %)

**Fig. 2:**
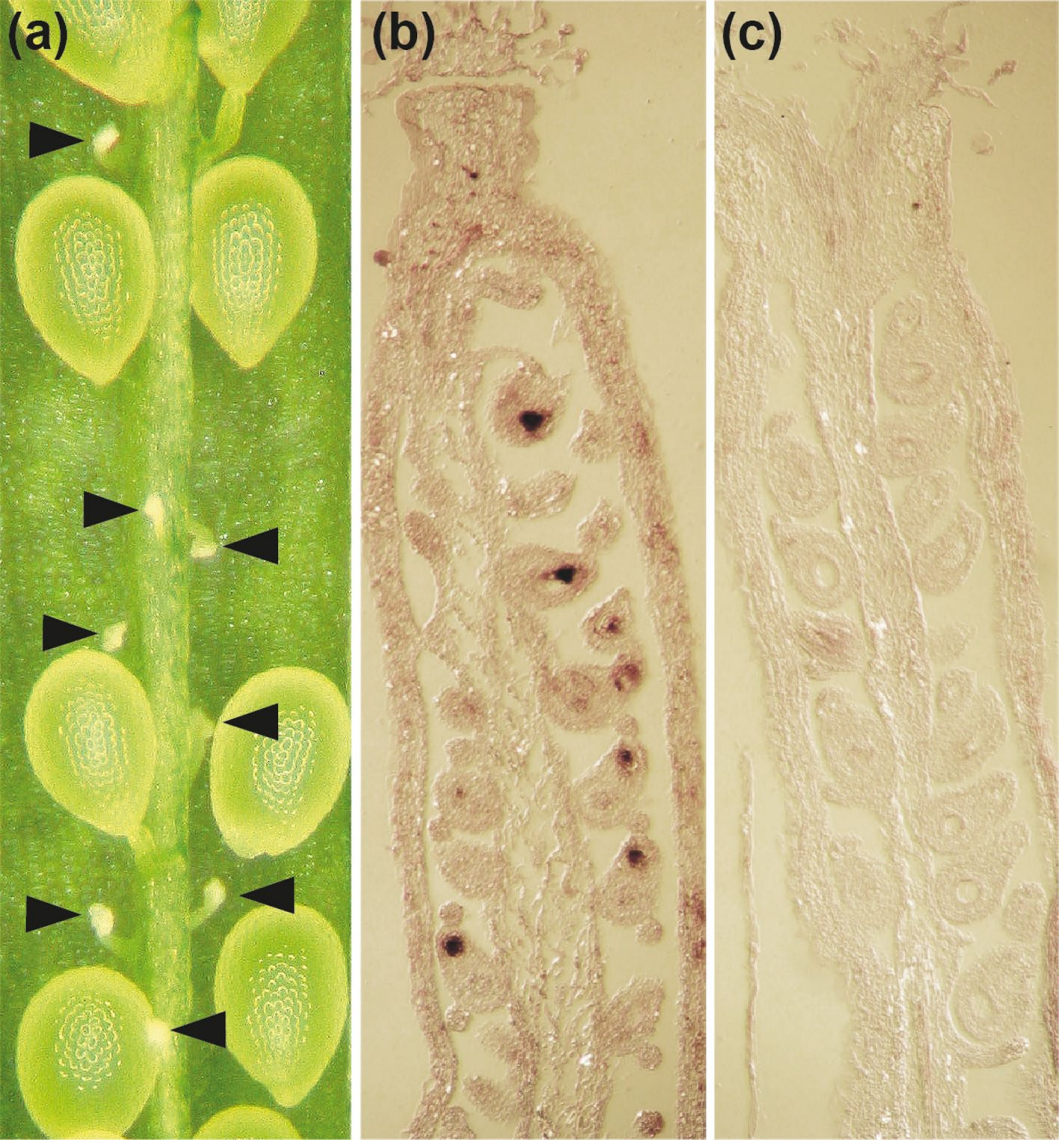
**a** Sterile ovules in siliques of the heterozygous mutant, indicated by *arrow heads*. In situ hybridization of carpel sections detecting HSFB2a mRNA with DIG labelled sense control (**b**) and antisense (**c**) probe

In order to determine the function of HSFB2a we analysed four different T-DNA insertion lines. GT12254, GT10826, and SALK_137766 contain insertions in the 5′-Nontranslated region, the intron, and the 3′ terminal coding region, respectively (Fig. 1a), and did not exhibit any defects. By contrast, for SALK_012418, which contains a T-DNA insertion in the middle of the first exon, we were not able to recover homozygous plants (n = 458). We designated the mutant allele *hsfB2a*-*tt1.* To better understand the mutant defect, we inspected the siliques of heterozygous *hsfB2a*-*tt1* plants. Notably, 44.9 % of all ovules (n = 3,461) were sterile as compared to 2.2 % in wild type (n = 3,740, Fig. 2a, Figure S1a), suggesting that the mutant was gametophytic lethal. In order to test whether the *hsfB2a*-*tt1* allele is passed on by both, male and female gametophytes, we conducted reciprocal crosses to Col-0 wild-type plants and found transmission rates of 21 and 22 % for female and male gametophytes, respectively (Table 1). Male transmission was substantially increased when the amount of pollen applied to the stigma was limited (Table 1), suggesting a reduced competitiveness of mutant pollen compared to wild-type.

**Table 1:** Genotypes and transmission rates of crossings

Genotypes F0	Genotype F1			
	*wt* (%)	*hsfB2a*-*tt1/*+ (%)	Transmission rate (%)	n
*hsfB2a*-*tt1/*+ × *hsfB2a*-*tt1/*+	77.7	22.3	44.6	1,446
*hsfB2a*-*tt1/*+ × *wt*	89.6	10.4	20.8	154
*wt* x *hsfB2a*-*tt1/*+	89.2	10.8	21.6	329
*wt* x *hsfB2a*-*tt1/*+^a^	65.4	34.6	69.2	107

^a^Reduced pollen

To characterize the female gametophytic defect associated with the reduced *hsfB2a*-*tt1* transmission, we performed cleared whole mounts of mutant and wild-type ovules 2 days after emasculation of the oldest closed flower bud, a stage where wild type gametophytes have undergone maturation. We found that 55.4 % of the gametophytes arrest before completion of all three mitotic divisions (Figure S1b). In 20.3 % of the arrested gametophytes four nuclei at the micropylar region of the gametophyte were visible (Figure S1b, Figure S2b). These results suggest that HSFB2a is required for the development of the female germline.

To determine whether the defect in *hsfB2a*-*tt1* was, indeed, due to reduced HSFB2a activity, we in a first step characterized the expression of the gene at control temperature (20 °C) and after heat shock (38 °C, 1 h). At 20° the expression in wild type and mutant was 2.7 and 1.1 % with respect to the internal standard *ACTIN2*. After heat-induction, expression reached a fourfold higher level in both lines (9.8 and 4.5 %) (Figure S3). These results confirm that *HSFB2a* is a heat-inducible gene and show that its activity is reduced by more than 50 % in *hsfB2a*-*tt1* mutants.

We next asked, whether *HSFB2a* could functionally complement the gametophytic lethal phenotype of *hsfB2a*-*tt1* and introgressed a genomic *HSFB2a* fragment, designated *HSFB2a*-*tg* into *hsfB2a*-*tt1.* We found only 4 (lines 75, 96, 165, 184) out of 189 BASTA resistant transformed plants in the T1 generation.

Microscopic inspection of female gametophytes of the T2 generation revealed an increase in the percentage of mature female gametophytes of 14.5 and 14.7 % in two out of four independent transgenic lines (lines 75 and 165, Figure S6). This small but significant improvement indicates that the female gametophytic defect relates to reduced *HSFB2a* activity, but also suggests that the expression levels conferred by *HSFB2a*-*tg* were not sufficient to reach those of the native *HSFB2a* allele.

Additionally, we tested two more lines with a T-DNA insertion in *HSFB2a* for an altered female gametophytic phenotype. These lines, GT10826 and GT12254, neither heterozygous nor homozygous mutant plants, showed a phenotype differing from wildtype. However, these lines are in a different genetic background (*Ler*) compared to *hsfB2a*-*tt1* (Col0) and the T-DNA insertions are located in the 5′-UTR (GT12254) and the intron (GT10826), respectively. In addition, we generated *HsfB2a*-mutants by generating overexpression lines for sense and antisense RNA (see below).

### Expression of *HSFB2a*

**Fig. 3:**
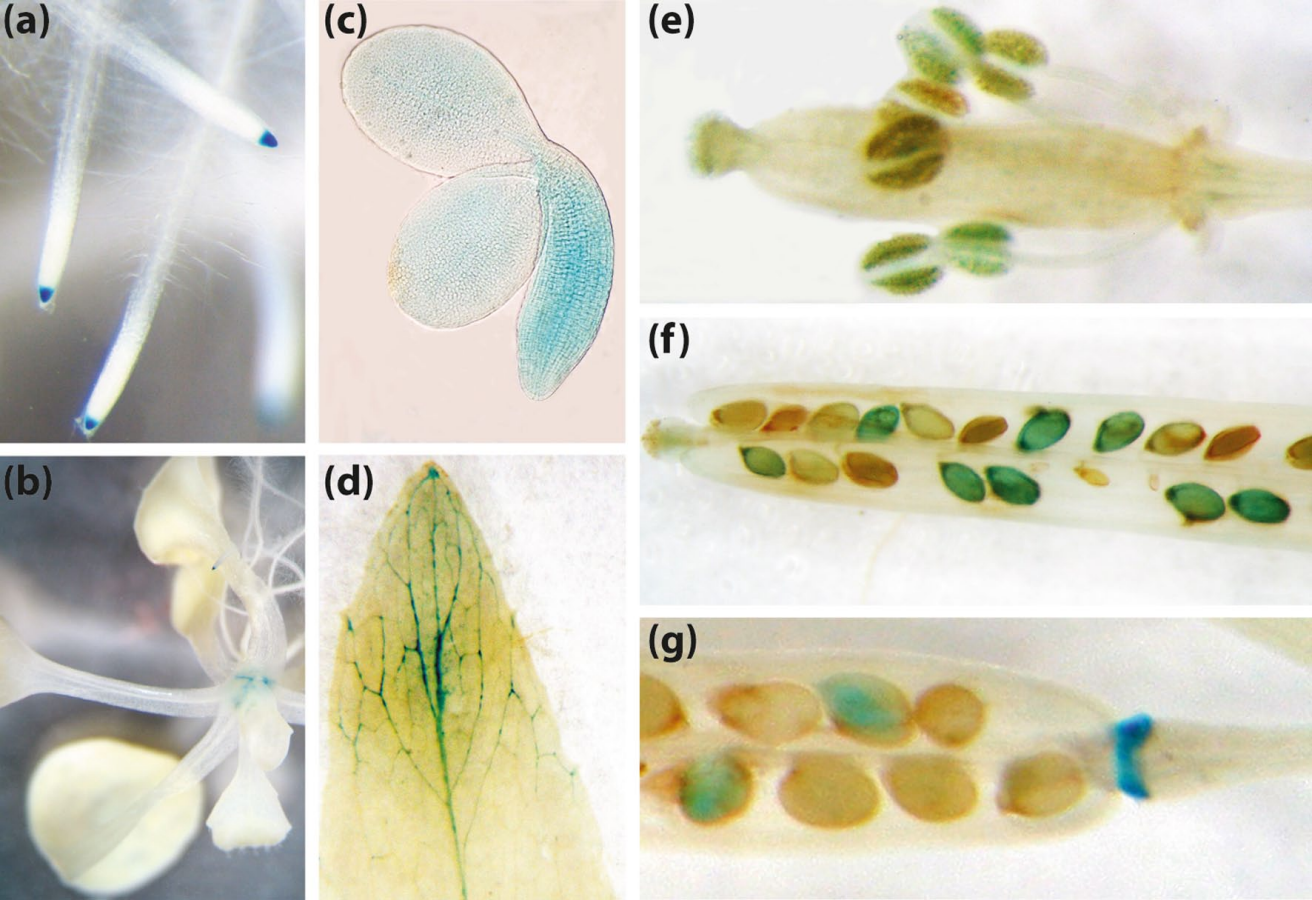
GUS expression in *HSFB2a*-*Promoter:GUS* plants. In roots (**a**) of 8 day old and nodes (**b**) of 15 day old seedlings, leaves (**d**) of 7 weeks old plants, anthers of flowers (**e**) of stage 13 (Smyth et al. 1990) and embryos (**c**), seed (**f**) and the junction of the petiole of ripe siliques (**g**)

In order to examine *HSFB2a* expression during early development we analysed promoter:GUS reporter expression in transgenic Arabidopsis plants. Using an 855 base pair (bp) DNA fragment upstream of the transcription start site of *HSFB2a* fused to the β-glucuronidase gene, we detected signals in the root tip and nodes of 8, respectively 15 day old seedlings (Fig. 3a, b). In the reproductive tissues we found staining in anthers of flowers at stage 13, according to Smyth et al. (1990), in walking stick embryos, and in the junction between receptacle and silique (Fig. 3c, e, f, g). At this stage, however, there was no staining detected in ovules. *GUS* expression in leaves was only detectable at the beginning of senescence in the vascular tissue (Fig. 3d).

### *HSFB2a* is expressed in the female gametophyte

Due to the gametophytic defect of *hsfB2A*-*tt1/*+ mutants we tested *HSFB2a* expression by in situ hybridization experiments. Sections of carpels were hybridized with a probe covering part of the 3′-UTR (Fig. 1a). We detected strong and specific hybridization signals in female gametophytes with the antisense probe, while no signal was obtained with the sense control probe (Fig. 2b, c).

By contrast, by using a probe covering the last 71 bp of the *HSFB2a* ORF and the first 40 bp of the 3′-UTR (Fig. 1a), hybridizations signals were not only obtained with the antisense probe, but an unprecedented strong signal was also detected in the sense probe control (Figure S4). These data suggest that in this part of the coding region, both strands of *HSFB2a* are transcribed. This interpretation is supported by the presence of an annotated cDNA clone, RAFL21-83-H09, with an 1,138 bp overlap with the genomic sequence of *HSFB2a*. This cDNA contains no ORF starting with an ATG and hence most probably is a non-coding RNA.

### Expression from the antisense strand of *HSFB2a*

The natural antisense RNA expression (*asHSFB2a*) at the *HSFB2*a locus, identified by in situ hybridization in the female gametophyte, was further analysed in leaves of wild-type plants. At different temperatures RNA levels were quantified by real-time qRT-PCR (Fig. 1c). The antisense RNA (*asHSFB2a*) was almost absent (0.01 % of *ACTIN* standard) at control temperature but was more than 1100-fold induced (11.5 % of *ACTIN* RNA standard) after one hour heat stress at 38 °C. Thus heat-induction of *asHSFB2a* RNA is more than 180-fold higher than that of *HSFB2a* mRNA, suggesting that not only *HSFB2a* but also *asHSFB2a* is a heat shock gene. We screened the *HSFB2a* and *asHSFB2a* upstream region for the presence of HSEs, which are characteristic to heat-inducible genes. In fact, in both putative promoter regions we found one perfect HSE with the consensus sequence nGAAnnTTCn located upstream of the transcriptional start sites of *HSFB2a* (22 bp) and *asHSFB2a* (71 bp) (Fig. 1a). The 1 kb upstream region of *asHSFB2a* contained, in addition, more than twice as many imperfect HSEs (with one bp change in consensus HSE) than the respective upstream region of *HSFB2a*.

The heat-induction of *HSFB2a* depends on the presence of class A heat shock factors HSFA1a and -A1b (Busch et al. 2005). This prompted us to examine also the *asHSFB2a* RNA levels in *hsfA1a/A1b* double knock-out plants (Busch et al. 2005). We found on average a 50-fold reduction of *asHSFB2a* RNA after heat shock as compared to wild type (Figure S7), indicating that also the antisense *HSFB2a* transcript is under regulatory control by class A heat shock factors.

### Manipulation of sense and antisense RNA expression

The strictly heat-inducible natural non-coding antisense RNA may specify a regulatory mechanism targeting the expression of specific genes. In order to test whether *HSFB2a* expression was affected by the antisense transcript *asHSFB2a* and vice versa we fused the CaMV 35S promoter to the full-length cDNA of *asHSFB2a* and *HSFB2a*. Transgenic Arabidopsis plants overexpressing either one of these constructs were examined by qRT-PCR for both, *HSFB2a* sense and *asHSFB2a* antisense expression at different temperatures. Compared with wild type, *35S:HSFB2a* plants showed a high constitutive level of the *HSFB2a* transcript, however, in these plants the *asHSFB2a* RNA was undetectable at all temperatures. Conversely, *35S:asHSFB2a* plants, expressing high constitutive levels of *asHSFB2a* RNA, did not express detectable *HSFB2a* mRNA levels at any temperature (Fig. 1b, c). These results indicate that *HSFB2a* and *asHSFB2a* are potent inhibitors of the respective other transcript. Together with our observation that *asHSFB2a* is massively induced upon heat stress, these results suggest that the noncoding *asHSFB2a* RNA buffers the HSFB2a-dependent heat shock response.

### *HSFB2a* links heat response and seedling size

**Fig. 4:**
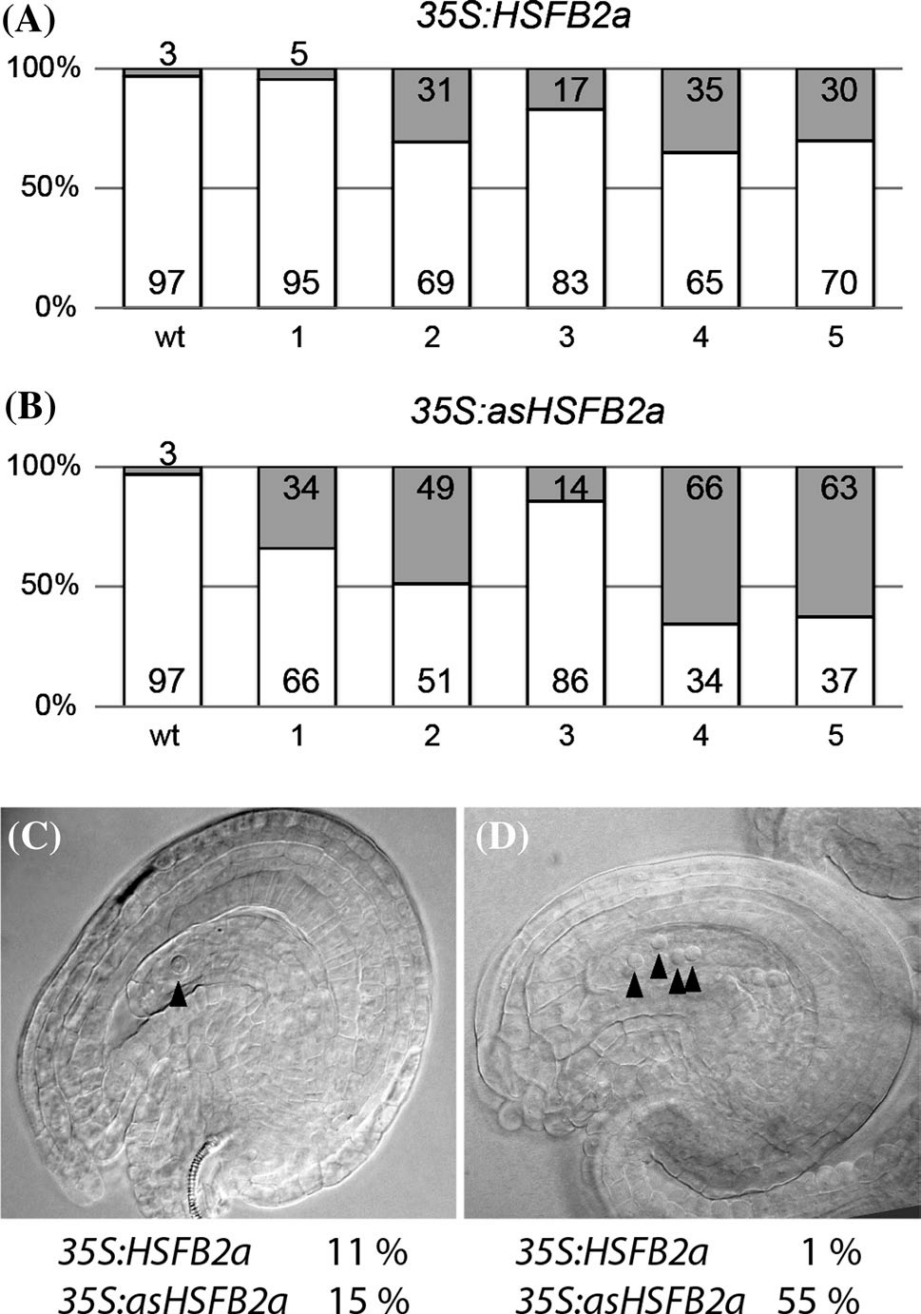
Proportions of mature and immature female gametophytes in (**A**) wt and five *HSFB2a* overexpressing (*35S:HSFB2a, 1*–*5*) and (**B**) wt and five *asHSFB2a* (*35S:asHSFB2a*, *1*–*5*) overexpressing lines. *Open boxes* represent the proportion of mature female gametophytes, *grey boxes* the proportion of immature female gametophytes, *numbers* in and above *boxes* the respective average percentage of five plants per line. Prevalent aberrant phenotypes of *HSFB2a* and *asHSFB2a* overexpressing lines. **C** Embryo sac with one nucleus. **D** Embryo sac with four nuclei. *Arrow heads* depict the position of visible nuclei in the female gametophytes. *Text* below the pictures indicates the percentage of gametophytes showing the above phenotype in the respective lines

The intriguing “Yin–Yang relationship” of RNA levels exhibited after transgenic manipulations of *HSFB2a* and as*HSFB2a* offers the opportunity to examine the effects of *HSFB2a* downregulation in the sporophyte by overexpressing as*HSFB2a.* For proof of concept, we in a first step examined the effect of as*HSFB2a*-mediated *HSFB2a* downregulation during ovule development. We found that 45 % of all ovules in *35S:*as*HSFB2a* lines exhibited an arrest in embryo sac development at various stages (Fig. 4a), reminiscent of the phenotype observed in *hsfB2A*-*tt1/*+ plants. Given that 35S activity is not detectable in the female gametophyte (Roszak and Köhler 2011), these data suggest that the amount of *asHSFB2a* in the sporophytic ovular tissue is decisive for the development of the female germline.

We next examined the effect of as*HSFB2a*-mediated *HSFB2a* downregulation in sporophytic tissue. Notably, in four independent *35S:asHSFB2a* antisense lines, we observed 10 days after germination an increase in biomass by 58 % as compared to wildtype. Conversely, plants overexpressing *HSFB2a* (*35S:HSFB2a*) exhibited a 15 % reduced fresh weight compared to wild type (Figure S5). These results indicate that *HSFB2a* has a negative impact on early plant growth and reveal an unprecedented link between the plant heat shock response and the regulation of seedling size.

## Discussion

### Regulation of *HSFB2a* and *asHSFB2a* expression

Using in situ hybridization and qRT-PCR *HSFB2a* mRNA was detected in female gametophytes and leaves respectively. In leaves heat-induced enhancement of transcript levels of *HSFB2a* have been described before (Busch et al. 2005), but surprisingly, our analyses revealed also a very strong and strictly heat stress dependent expression of a long non-coding antisense RNA. This gene, *asHSFB2a*, is transcribed in opposite orientation starting shortly beyond the 3′-end of the HSFB2a coding region and terminating way upstream of the 5′-end of the *HSFB2a* gene. In leaves the heat-induction of *asHSFB2a* is much stronger than of *HSFB2a* mRNA. Differences in strength of heat-induction correlate with the differences in the numbers of imperfect HSEs in the respective 1 kb promoter upstream regions. It has been shown that heat-induced *HSFB2a* expression is controlled by HSFA1a/HSFA1b (Busch et al. 2005). Our re-examination of the same RNA preparations clearly indicated that the *asHSFB2a* expression is also dependent on HSFA1-HSFs, which are known to control the majority of early induced heat shock genes (Yoshida et al. 2011). A regulatory function of *asHSFB2a* RNA on *HSFB2a* expression was verified by transgenic overexpression. CaMV 35S promoter-driven *asHSFB2a* expression resulted in a complete shut off of *HSFB2a* expression at all temperatures, suggesting an effective antisense effect in leave tissue. It should be noted that in the leaves of native plants the *asHSFB2a* antisense effect can be only implemented after heat stress, significant levels are only reached at temperatures above 34 °C.

Interestingly, *CaMV 35S*-driven *HSFB2a* overexpression imposes also a strong negative effect on the level of *asHSFB2a* RNA. Under these conditions the sense RNA acts as a “silencing antisense RNA” on *asHSFB2a* expression. The molecular mechanism of this “Yin–Yang” control of sense and antisense RNA expression is unknown. Long non-coding RNAs may cause transcriptional silencing through promoting the formation of chromatin-modifying complexes (Beisel et al. 2007; Schuettengruber et al. 2007; Shilatifard 2008; Wang and Chang 2011). In Arabidopsis non-coding sense transcripts termed *COLDAIR*, originating from the first intron of FLC, have recently been shown to be sufficient *to maintain repression of* FLC during vernalization (Helliwell et al. 2011). The *COOLAIR* antisense transcripts (Swiezewski et al. 2009) originating from a promoter adjacent to the *FLC* 3′ untranslated region seems to have only a redundant function in silencing the FLC locus.

Is *asHSFB2a*-dependent regulation of *HSFB2a* also implemented in the female gametophyte? By in situ hybridization *asHSFB2a* RNA was detected in ovules. Manipulation of *HSFB2a* and respectively *asHSFB2a* expression via 35S-promoter-driven transgenes resulted in significant changes in the proportions of defective gametophytes (Fig. 4). These defective phenotypes are overall more pronounced in *asHSFB2a* transgenic lines, which is in support of the detrimental effect of the *hsfB2a*-*tt1* mutation. There is some variation in the proportions of defective ovules, but at present it is unknown how strong constitutive 35S-promoter-driven gene expression is implemented in cells of the gametophyte. It is not clear whether the 35S-promoter is fully active at all stages during female gametophyte development in Arabidopsis (Jiang et al. 2010; Liu et al. 2008; Roszak and Köhler 2011). Our observation that *35S*-driven expression of *asHSFB2a* interferes with female gametophyte development might relate to the fact that the unicellular embryo sac inherits its cytoplasm from sporophytic, i.e. *asHSFB2a* containing tissue. Alternatively, *HSFB2a* is required sporophytically to ensure proper female gametophyte development. Our data suggest that changes in *HSFB2a* expression levels are crucial for proper female gametophyte development. Our finding that the female gametophyte and seed defects of *hsfB2a*-*tt1/*+ could only be rescued to a minor extend by a genomic *HSFB2a* fragment might hint to a lack of critical *cis*-regulatory elements in the sequence provided.

### Growth phenotype of *HSFB2a* and *asHSFB2a* overexpression lines

The 35S-driven overexpression of *HSFB2a* and *asHSFB2a* in transgenic lines had clear effects on the gametophyte and on the plant growth phenotype. The *asHSFB2a*-ox lines showed an improved, the *HSFB2a*-ox lines an impaired biomass production, but only in the early phase of plant growth. This suggests that during development HSFB2a activity temporarily represses vegetative growth and following heat stress the antisense regulation by *asHSFB2a* counteracts this effect to restore growth and further development.

### HSFB2a promotes nuclear proliferation in the female gametophyte

Characteristic to gametophytic mutants is the non-Mendelian inheritance of mutant alleles, which is also referred to as segregation distortion (Drews et al. 1998; Drews and Yadegari 2002; Moore et al. 1997; Page and Grossniklaus 2002). The *hsfB2a*-*tt1/*+ plants, heterozygous for the heat shock factor HSFB2a, exhibit 50 % sterile ovules and a substantially reduced male and female transmission, indicating that the absence of the gene is detrimental to the development of the male and female germ lines. The finding that male transmission is enhanced by reducing the amount of pollen applied to the pistil suggests that the defect is at least partially due to retarded pollen development or tube growth.

The analysis of *hsfB2a*-*tt1* female gametophytes revealed an impaired development during megagametogenesis. Most of the phenotypically abnormal ovules exhibited four nuclei of similar size and the crosses of *hsfB2a*-*tt1/*+ with the egg cell marker line *DD45::NLS_GUS* provided no evidence for the specification of an egg cell in the arrested gametophytes. Notably, a small proportion of *hsfB2a*-*tt1* female gametophytes gave rise to functional seeds when pollination was delayed, reminiscent to results gained for *swa1*–*swa3* mutants (Li et al. 2009; Liu et al. 2010; Shi et al. 2005). *Swa1*–*3* mutants are defective in different rRNA processing factors and exhibit retarded division cycles in the female gametophyte. Whether HSFB2a can be linked to the biogenesis of ribosomal RNA is currently unclear.

## Experimental procedures

### Plant materials and growth conditions

*Arabidopsis thaliana* accession Columbia (Col-0) was used as the wild type. Seeds of SALK_012418 were obtained from the Nottingham Arabidopsis Stock Centre, seeds of GT10826 and GT12254 were provided by Robert Martienssen (Cold Spring Harbor Laboratory).

*S*eeds were sown in soil, kept at 4 °C for 2 days in the dark and grown in a chamber under white fluorescent light (80 μmol m^−2^ s^−1^) with a 16 h/8 h light/dark cycle at 20 °C and 60 % relative humidity. For selection of mutant plants, seeds were surface-sterilized and sown on plates containing Murashige & Skoog medium including vitamins with 0.8 % (w/v) phyto agar (both DUCHEFA, Haarlem, Netherlands) supplemented with 2 % (w/v) sucrose and 50 µg/ml Kanamycin. Following cold treatment at 4 °C for 2 days in the dark, plates were incubated under standard growth conditions. After 10 days, resistant plants were transferred to soil. Transgenic lines carrying a phosphinothricin resistance gene were grown on soil and for selection sprayed with 0.1 % (v/v) BASTA (AgrEvo, Germany) 8–10 days after germination.

For mRNA analysis two leaves from each of 20 five-week-old plants were incubated for 1 h at the respective temperature in SIB-puffer (1 mM potassium phosphate, pH 6.0, 1 % sucrose) in the dark in a shaking water bath. After treatment leaves were immediately frozen in liquid nitrogen and stored at −80 °C.

### DNA constructs and the generation of transgenic plants

All PCR reactions for cloning were conducted with PHUSION polymerase (New England Biolabs) and correctness of the clones was confirmed by sequencing. For complementation of *hsfB2a*-*tt1/*+ plants a 3,418 bp genomic fragment spanning the *HSFB2a* sequence with 1,180 bp upstream of the transcription start site and 684 bp downstream of the 3′-UTR was cloned by restriction of P1 clone MITG10 (Liu et al. 1995), obtained from the Arabidopsis Biological Resource Center, (http://abrc.osu.edu/) with *Spe*I and *Msc*I and insertion of the fragment into *Spe*I/*Sma*I of the binary vector pCB308 (Xiang et al. 1999).

For *HSFB2a* overexpression in plants a 1,555 bp fragment was amplified by PCR from clone MITG10 with the primers B2aOXFKpnI and B2aOXRSacI and joined via *Sac*I to a 813 bp CaMV promoter sequence that was amplified from pBI121 (Chen et al. 2003) with the primers CamVSacF2 and CaMVKpnR2. The resulting construct was inserted into *Sac*I of pCB308. For *asHSFB2a* overexpression in plants a 1,458 bp fragment with the complete sequence of the annotated cDNA clone RAFL21-83-H09 was amplified by PCR from clone MITG10 with the primers ASFKpnI and ASRSacI and joined via *Sac*I to the CaMV promoter sequence as above. The resulting construct was inserted in *Sac*I of pCB308. For β-glucuronidase (GUS) expression under the control of the *HSFB2a* promoter a 949 bp fragment comprising 855 bp upstream of the transcription start site and 94 bp of the 3′-UTR of *HSFB2a* was amplified with *Xba*I recognition site containing primers B2aPromF and B2aPromR. There were no relevant promoter elements detectable in the extra 300 bp region of the 1,180 bp promoter (used for complementation, see above) compared to the 855 bp promoter. After digestion of the PCR product with *Xba*I the fragment was inserted in the *Xba*I site in pCB308. The respective constructs were introduced into *Agrobacterium tumefaciens* strain GV3101 by electroporation and transgenic plants were created by the vacuum infiltration method (Bechtold et al. 1993).

### Southern blot hybridization

To rule out a second insertion elsewhere in the genome, we digested genomic DNA of the heterozygous mutant, segregating plants with no T-DNA insertion in *HSFB2a* and Col0 wild type with *Hin*dIII and subjected it to southern blot hybridization probed with a radioactively labeled 203 bp fragment amplified with the primers TDNALBF/TDNALBR, specific for the left border sequence of the T-DNA. Two specific bands were detected only in the digest of the heterozygous mutant, not in wild type segregants.

### Isolation of mRNA and quantitative real-time RT-PCR

RNA was isolated from 100 mg frozen leaf powder with the CHEMAGIC mRNA Direct Kit (Chemagen, Germany). Reverse transcription was performed with one fifth of the eluted mRNA using the ISCRIPT cDNA Synthesis Kit (Biorad, Hercules, CA).

Quantitative PCR reactions were performed using SYBR^®^ Green technology on the ICYCLER system (Biorad, Hercules, CA). *HSFB2a* cDNA was amplified with primers HSFB2AF2 and HSFB2AR2, *asHSFB2a* cDNA with the primers ASB2AF and ASB2AR, ACTIN2 cDNA with the primers ACTIN2F3 and ACTIN2R3. For each genotype, at least three independent biological replicates were analysed in triplicate PCR reactions. Relative expression of transcripts was quantified with respect to *Actin2* (At3g18780) as internal standard.

### Determination of 5′ and 3′-ends of mRNA

To define transcriptional start and termination sites of *asHSFB2a* a 5′- and 3′-RACE was performed by using the Invitrogen GeneRacer^®^ Kit with SuperScript^®^ III RT and TOPO TA Cloning^®^ Kit for sequencing. Following the protocol of the manufacturer we used mRNA from heat shocked (1 h, 38 °C) wild-type leaves and the primers ASGSP5′RACE and ASGSP3′RACE as the respective 5′- and 3′-specific primers for amplification and sequencing. Nine and eight clones of the PCR products for the 5′-end and the 3′-end, respectively, were sequenced and the resulting nucleotide sequences compared to the TAIR annotation of the RIKEN cDNA clone RAFL21-83-H09. The 5′ end of seven clones was identical or 2–3 nucleotides shorter than the TAIR sequence, while two clones showed a 52 and 51 nucleotide shorter sequence, respectively. The 3′-end of five PCR clones was the same or within a range of five nucleotides as the annotated sequence. Three PCR products were truncated in a range of 32–181 nucleotides.

### In situ hybridisation

Flowers of stage 13 and 14 (Smyth et al. 1990) were harvested into FAA solution (3.7 % formaldehyde, 50 % ethanol, 5 % acetic acid) and embedded in paraplast. Sections of eight μm thickness were prepared with a microtome (Leica Biocut 2,035) and transferred on microscope slides (SUPERFROST ULTRA PLUS, Thermo Scientific). Probes were synthesized with the DIG RNA Labelling Kit (Roche) on a PCR fragment of *HSFB2a* generated with primers SP6-2B2aR2 and T7-B2aF2 (*HSFB2a*-specific) or with primers HSFB2aF2 and HSFB2aR2. Hybridization at 50 °C and detection were carried out as described by (Wollmann et al. 2010).

### Crossings and genotyping of T-DNA lines

Wild type and insertion mutants were emasculated 2 days before anthesis and cross-pollinated 2 days later. Crosses with limited pollen were conducted by transferring mature pollen of *hsfB2a*-*tt1/*+ anthers to a wild type stigma with the help of a ciliary. SALK_012418 and the progeny of the respective crossings were genotyped for the *hsfB2A*-*tt1* allele using T-DNA specific primer LBa1 and either of the gene specific primers N512418R or N512418L, which amplify the wild type allele. PCR products were sequenced to confirm the insertion site of the T-DNA after nucleotide 260 of the TAIR annotated cDNA. On both ends of the insertion we found the left border sequence of the T-DNA. To rule out a second insertion elsewhere in the genome, we digested genomic DNA of the heterozygous mutant, segregating plants with no T-DNA insertion in *HSFB2a* and Col0 wild type with *Hin*dIII and subjected it to southern blot hybridization probed with a radioactively labeled 203 bp fragment amplified with the primers TDNALBF/TDNALBR, specific for the left border sequence of the T-DNA. Two specific bands were detected only in the digest of the heterozygous mutant, not in wild type segregants.

### Microscopy and staining of ovules

For microscopy of female gametophytes the oldest closed flower bud of an inflorescence was emasculated and harvested 2 days later. Whole-mount clearings of ovules were performed as described (Yadegari et al. 1994). Cleared whole-mounts were visualized using a Zeiss AXIOSCOP Microscope (Zeiss, Oberkochen, Germany).

### Seed analysis in *hsfB2A*-*tt1/*+ heterozygotes and wild type

For seed counting siliques at position no. 6-10 of the primary inflorescence were placed on double-sided tape on a microscope slide and opened with a scalpel under a dissecting microscope.

## Electronic supplementary material

Below is the link to the electronic supplementary material.

Supplementary material Figure S1: Seed content and female gametophytic phenotype of mutant *hsfB2a-tt1/+* and wild-type plants. (a) Average number of seeds/silique in heterozygous mutant *hsfB2a-tt1/+* and wild-type plants. Error bars show standard deviation, number of plants ≥10, siliques/plant ≥5. (b) Female gametophytic phenotype of wild-type (top) and mutant (bottom) ovules; SN synergid cell nucleus, EN egg cell nucleus, CN central cell nucleus. Arrow heads depict the position of visible nuclei in the female gametophytes. (TIFF 7053 kb)

Supplementary material Figure S2: Phenotypes of female gametophytes in ovules. Distributions in (a) wild-type (n= 641) and (b) *hsfB2a-tt1* (n=960) plants. Numbers are percentage of all gametophytes in the respective category. In *hsfB2a-tt1* plants 4.4 % of the ovules contained three nuclei, two nuclei were observed in 12.9 % of all cases, and only one nucleus was present at 5.2 %. Empty ovules, without a nucleus, were present at 11.6 % in *hsfB2a-tt1/+* as compared to 3.9 % in wild type. Other immature stages (one or two nuclei) are negligible in wild type. (TIFF 12295 kb)

Supplementary material Figure S3: Expression levels of *HSFB2a* in *hsfA1a/hsfA1b*. Levels of mRNA were determined at control temperature (20°C) and after heat shock (38°C) in wt, heterozygous mutant plants (*hsfB2a-tt1*/+), and plants with an additional transgenic copy of *HsfB2a (HsfB2a-tt1/+/HSFB2a-tg 75, -96, -165, -184)*. Relative qRT-PCR levels were normalized with respect to Actin2 mRNA (= 100 %). Error bars indicate standard deviation (n=3). (TIFF 7643 kb)

Supplementary material Figure S4: *In situ* hybridization with an unspecific probe. Carpel sections were hybridized with DIG labelled sense (a) and antisense probe (b) derived from the overlapping region of *HSFB2a* and *asHSFB2a.* (TIFF 4156 kb)

Supplementary material Figure S5: Fresh weight of 35S:*HSFb2a*-ox and 35S:*asHSFb2a*-ox seedlings. Numbers refer to the respective lines. Bars represent the average relative fresh weight of 30 ten day old seedlings. Error bars indicate standard deviation (n=3). Asterisks indicate a significant difference between wild-type and the transgenic line (P<0.05). (TIFF 8450 kb)

Supplementary material Figure S6: Partial rescue of the gametophytic arrest with an extra copy of *HSFB2a*. Fractions ( %) of mature female gametophytes in ovules of plants with the indicated genotype are shown. *HSFB2a*-*tt1*/+*/HSFB2a*-*tg*: heterozygous mutant plants with an additional transgenic copy of *HSFB2a*. Error bars indicate standard deviation (plants per line ≥5). Asterisks indicate a significant difference between *hsfB2a-tt1/+* and transgenic lines *hsfB2a-tt1*/+/*HSFB2a-tg* 75, 165, 184 (* p<0.02, ** p<0.0001). (TIFF 5635 kb)

Supplementary material Figure S7: Antisense expression in *hsfA1a/hsfA1b* plants. Levels of mRNA were determined at control temperature (20°C) and after heat shock (38°C) in wt and *hsfA1a/hsfA1b* plants. Relative qRT-PCR levels were normalized with respect to Actin2 mRNA (= 100 %). (TIFF 4325 kb)

Supplementary material Figure S8: Southern blot hybridization for T-DNA.Genomic DNA of heterozygous mutant (*hsfB2a-tt1/+*), wild-type plants and the segregating progeny of *hsfB2a-tt1/+* with no T-DNA insertion in *HSFB2a* (*HSFB2a/HSFB2a* 1-6) was digested with *Hin*dIII and the gel blot was probed with a T-DNA specific fragment. Numbers on the left indicate the position of DNA marker bands in kbp. (TIFF 3279 kb)

Supplementary material 9 (DOCX 14 kb)
