# Supplementary figures and images for: Heat shock factor HSFB2a involved in gametophyte development of Arabidopsis thaliana and its expression is controlled by a heat-inducible long non-coding antisense RNA
Source: Plant Mol Biol. 2014 May 30;85(6):541–50. doi: 10.1007/s11103-014-0202-0 (PMC4099531; doi:10.1007/s11103-014-0202-0)

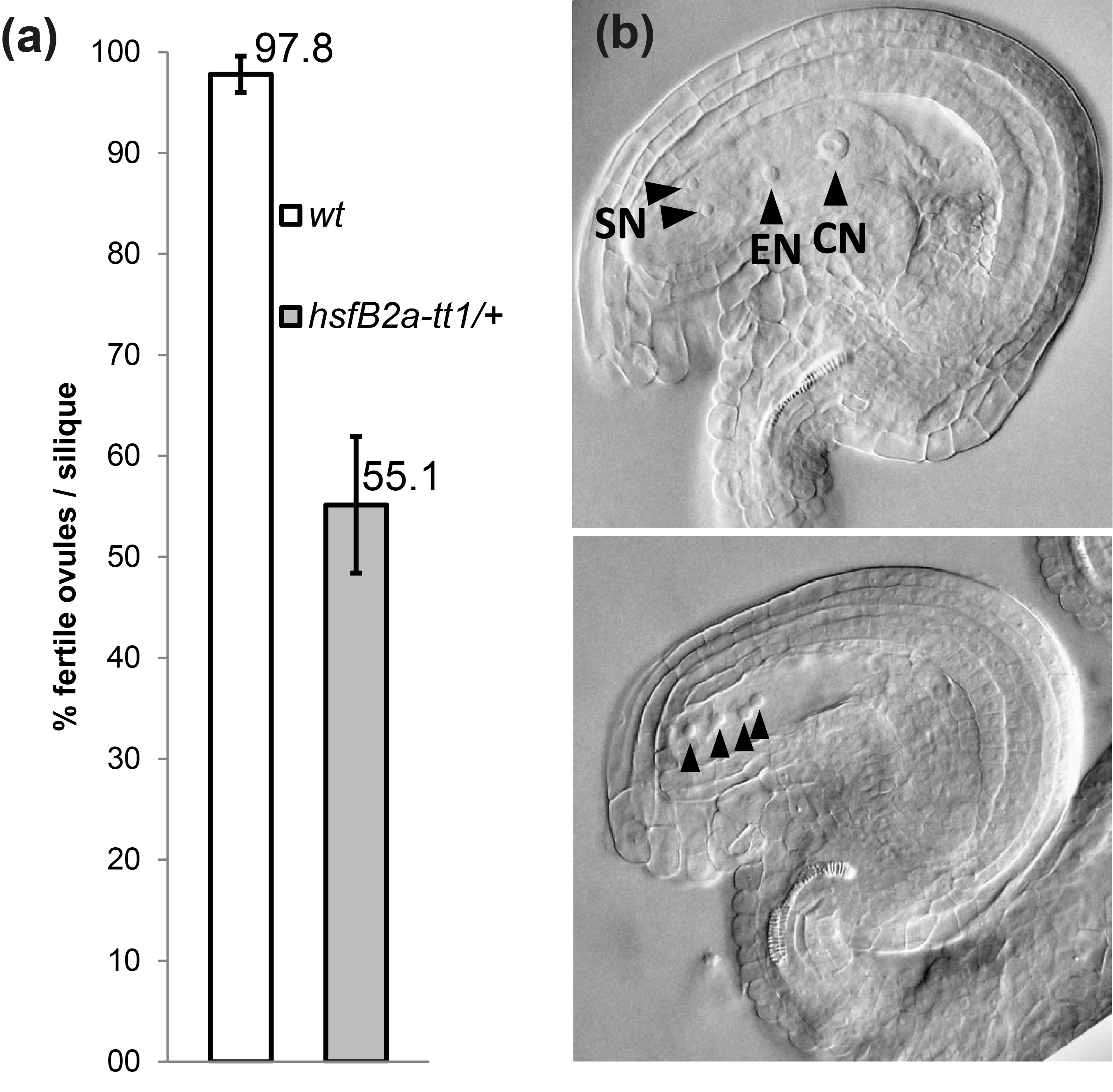

Supplement: Supplementary file 1 — Supplementary material Figure S1: Seed content and female gametophytic phenotype of mutant hsfB2a-tt1/+ and wild-type plants. (a) Average number of seeds/silique in heterozygous mutant hsfB2a-tt1/+ and wild-type plants. Error bars show standard deviation, number of plants ≥10, siliques/plant ≥5. (b) Female gametophytic phenotype of wild-type (top) and mutant (bottom) ovules; SN synergid cell nucleus, EN egg cell nucleus, CN central cell nucleus. Arrow heads depict the position of visible nuclei in the female gametophytes. (TIFF 7053 kb) [file 11103_2014_202_MOESM1_ESM.tif]

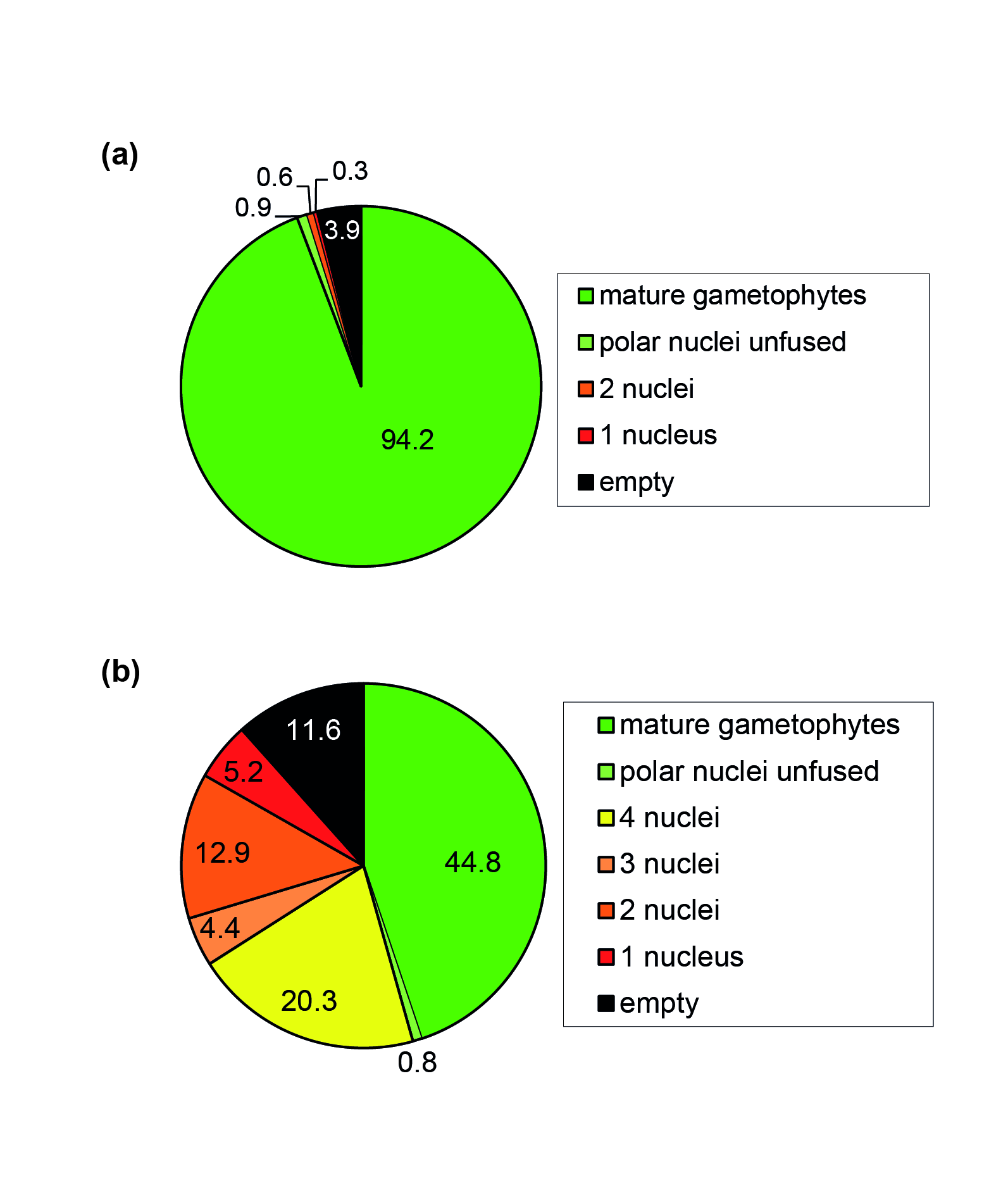

Supplement: Supplementary file 2 — Supplementary material Figure S2: Phenotypes of female gametophytes in ovules. Distributions in (a) wild-type (n= 641) and (b) hsfB2a-tt1 (n=960) plants. Numbers are percentage of all gametophytes in the respective category. In hsfB2a-tt1 plants 4.4 % of the ovules contained three nuclei, two nuclei were observed in 12.9 % of all cases, and only one nucleus was present at 5.2 %. Empty ovules, without a nucleus, were present at 11.6 % in hsfB2a-tt1/+ as compared to 3.9 % in wild type. Other immature stages (one or two nuclei) are negligible in wild type. (TIFF 12295 kb) [file 11103_2014_202_MOESM2_ESM.tif]

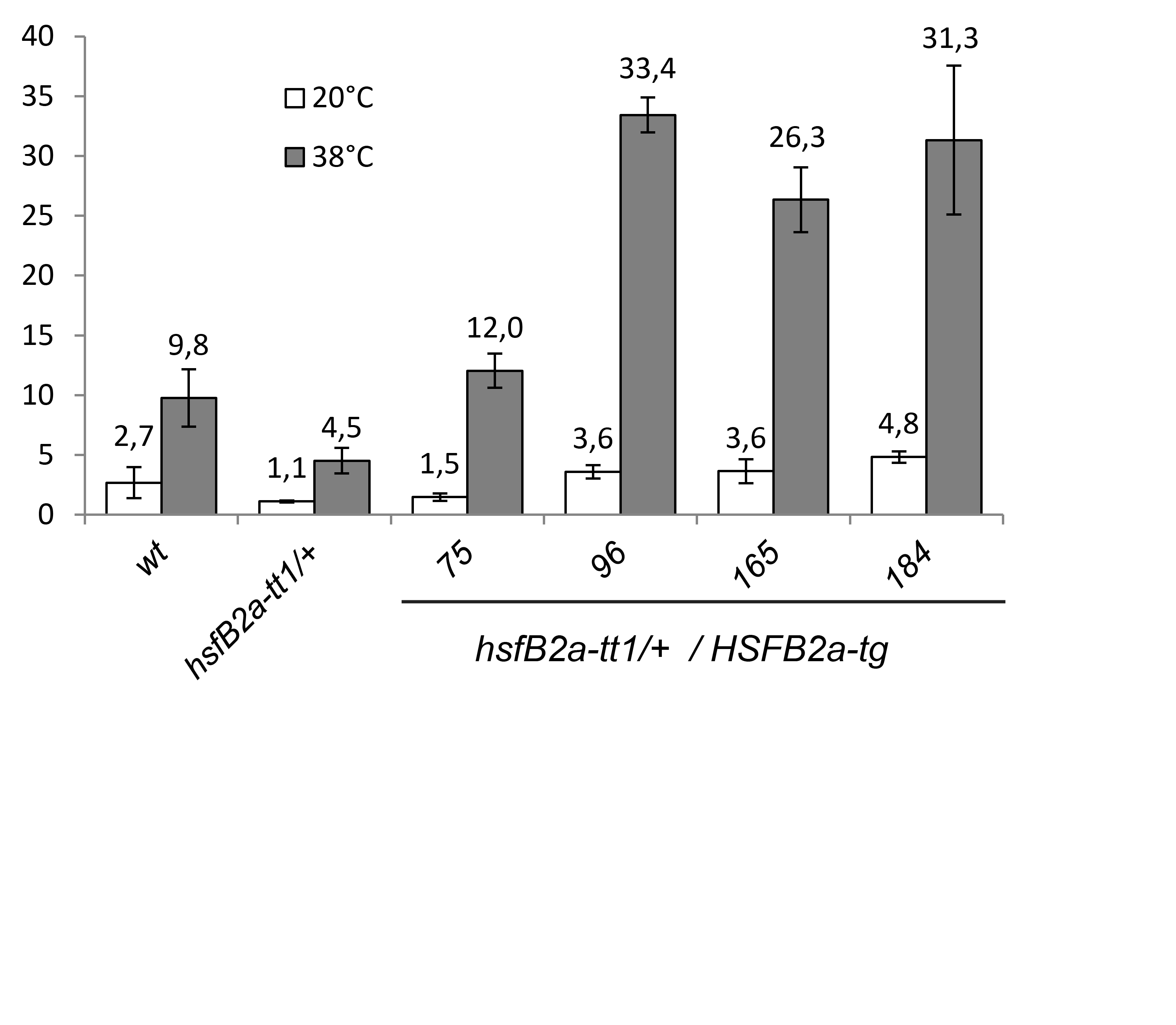

Supplement: Supplementary file 3 — Supplementary material Figure S3: Expression levels of HSFB2a in hsfA1a/hsfA1b. Levels of mRNA were determined at control temperature (20°C) and after heat shock (38°C) in wt, heterozygous mutant plants (hsfB2a-tt1/+), and plants with an additional transgenic copy of HsfB2a (HsfB2a-tt1/+/HSFB2a-tg 75, -96, -165, -184). Relative qRT-PCR levels were normalized with respect to Actin2 mRNA (= 100 %). Error bars indicate standard deviation (n=3). (TIFF 7643 kb) [file 11103_2014_202_MOESM3_ESM.tif]

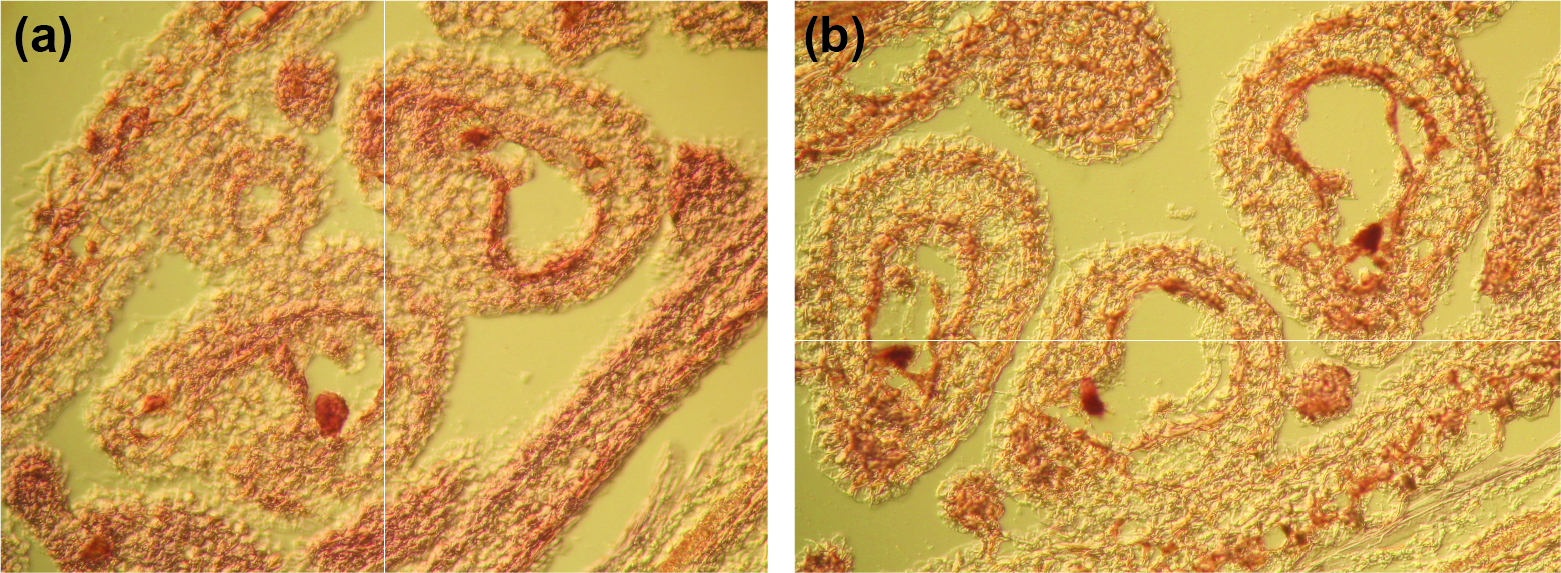

Supplement: Supplementary file 4 — Supplementary material Figure S4: In situ hybridization with an unspecific probe. Carpel sections were hybridized with DIG labelled sense (a) and antisense probe (b) derived from the overlapping region of HSFB2a and asHSFB2a. (TIFF 4156 kb) [file 11103_2014_202_MOESM4_ESM.tif]

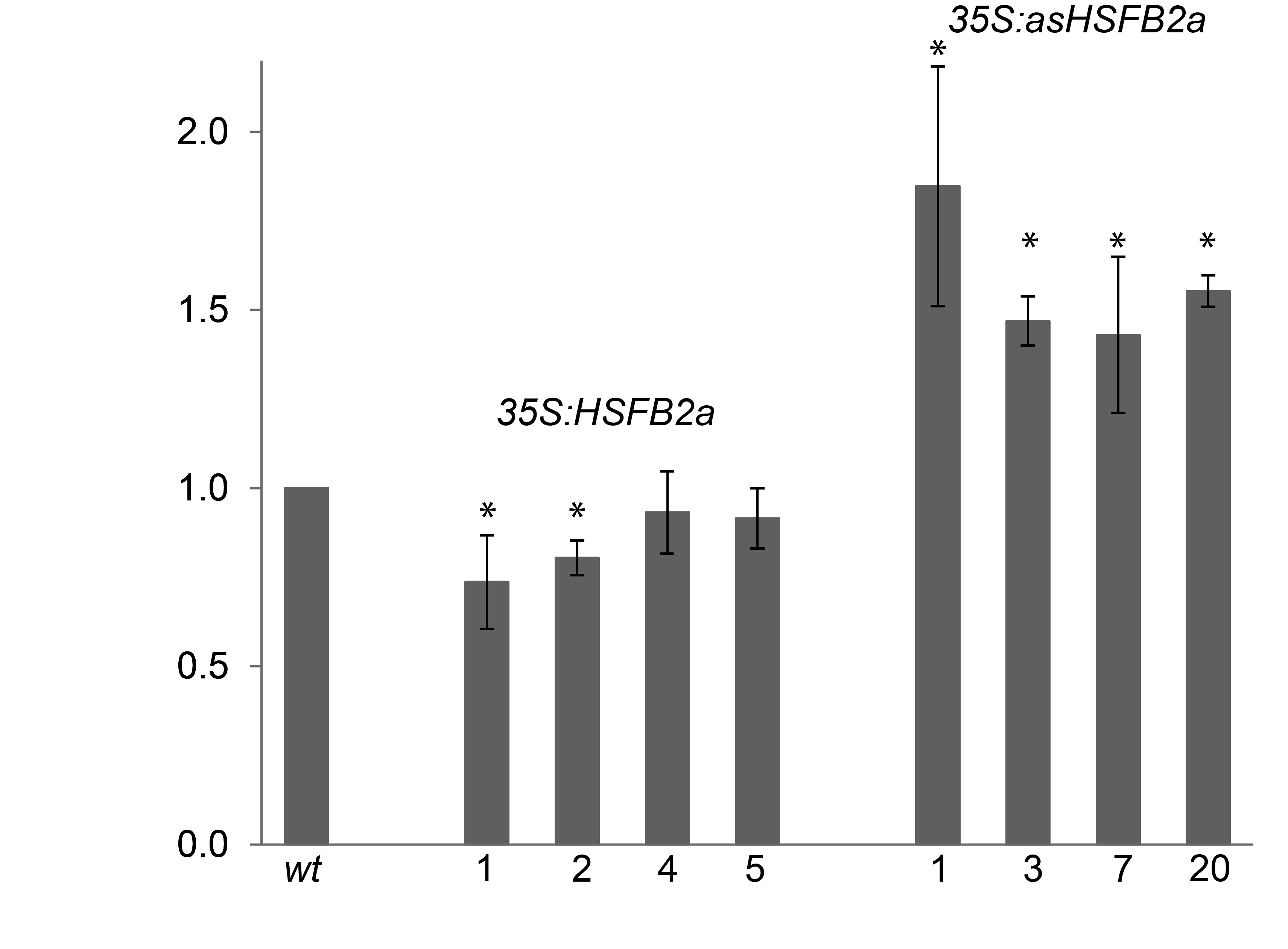

Supplement: Supplementary file 5 — Supplementary material Figure S5: Fresh weight of 35S:HSFb2a-ox and 35S:asHSFb2a-ox seedlings. Numbers refer to the respective lines. Bars represent the average relative fresh weight of 30 ten day old seedlings. Error bars indicate standard deviation (n=3). Asterisks indicate a significant difference between wild-type and the transgenic line (P<0.05). (TIFF 8450 kb) [file 11103_2014_202_MOESM5_ESM.tif]

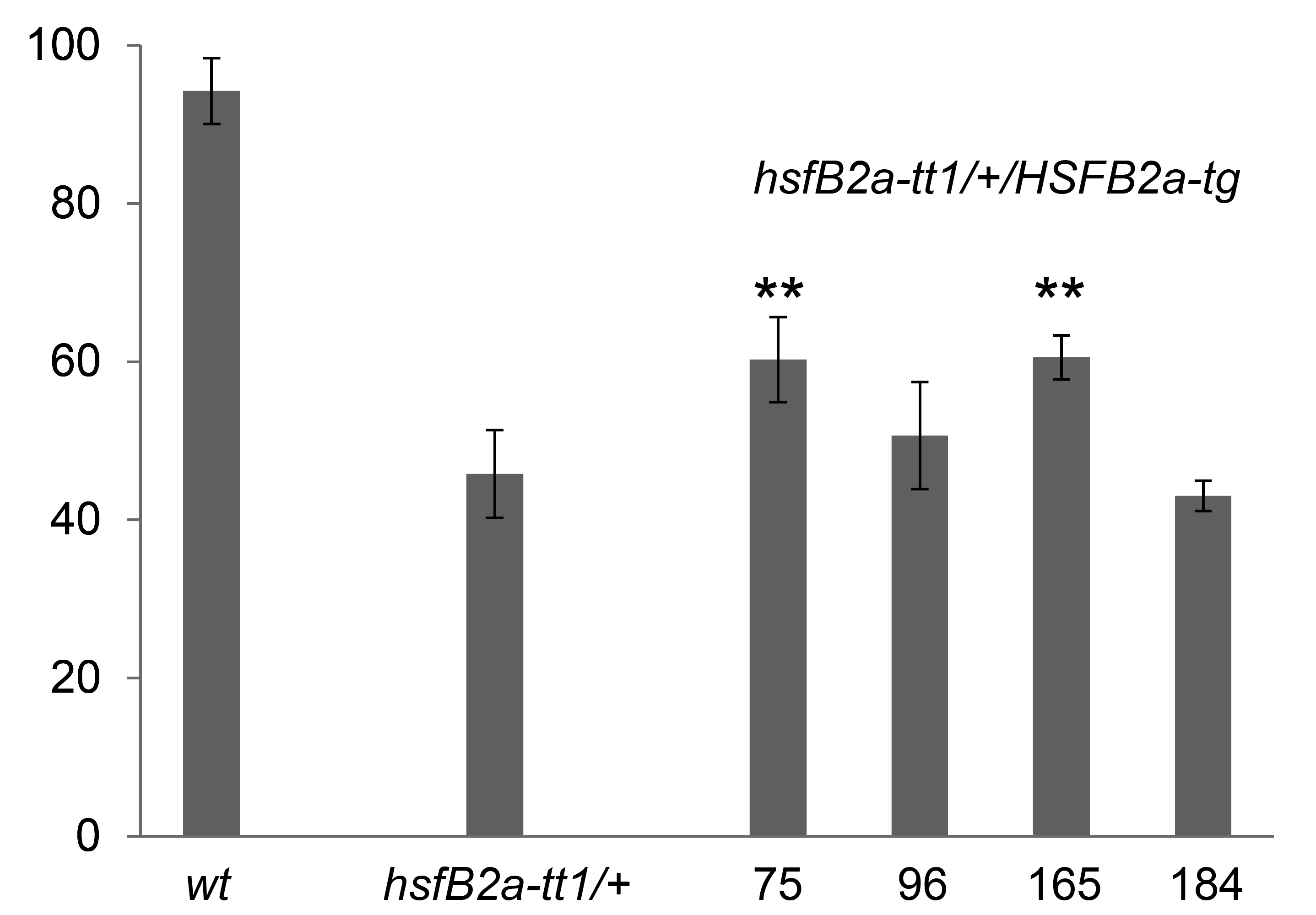

Supplement: Supplementary file 6 — Supplementary material Figure S6: Partial rescue of the gametophytic arrest with an extra copy of HSFB2a. Fractions ( %) of mature female gametophytes in ovules of plants with the indicated genotype are shown. HSFB2a-tt1/+/HSFB2a-tg: heterozygous mutant plants with an additional transgenic copy of HSFB2a. Error bars indicate standard deviation (plants per line ≥5). Asterisks indicate a significant difference between hsfB2a-tt1/+ and transgenic lines hsfB2a-tt1/+/HSFB2a-tg 75, 165, 184 (* p<0.02, ** p<0.0001). (TIFF 5635 kb) [file 11103_2014_202_MOESM6_ESM.tif]

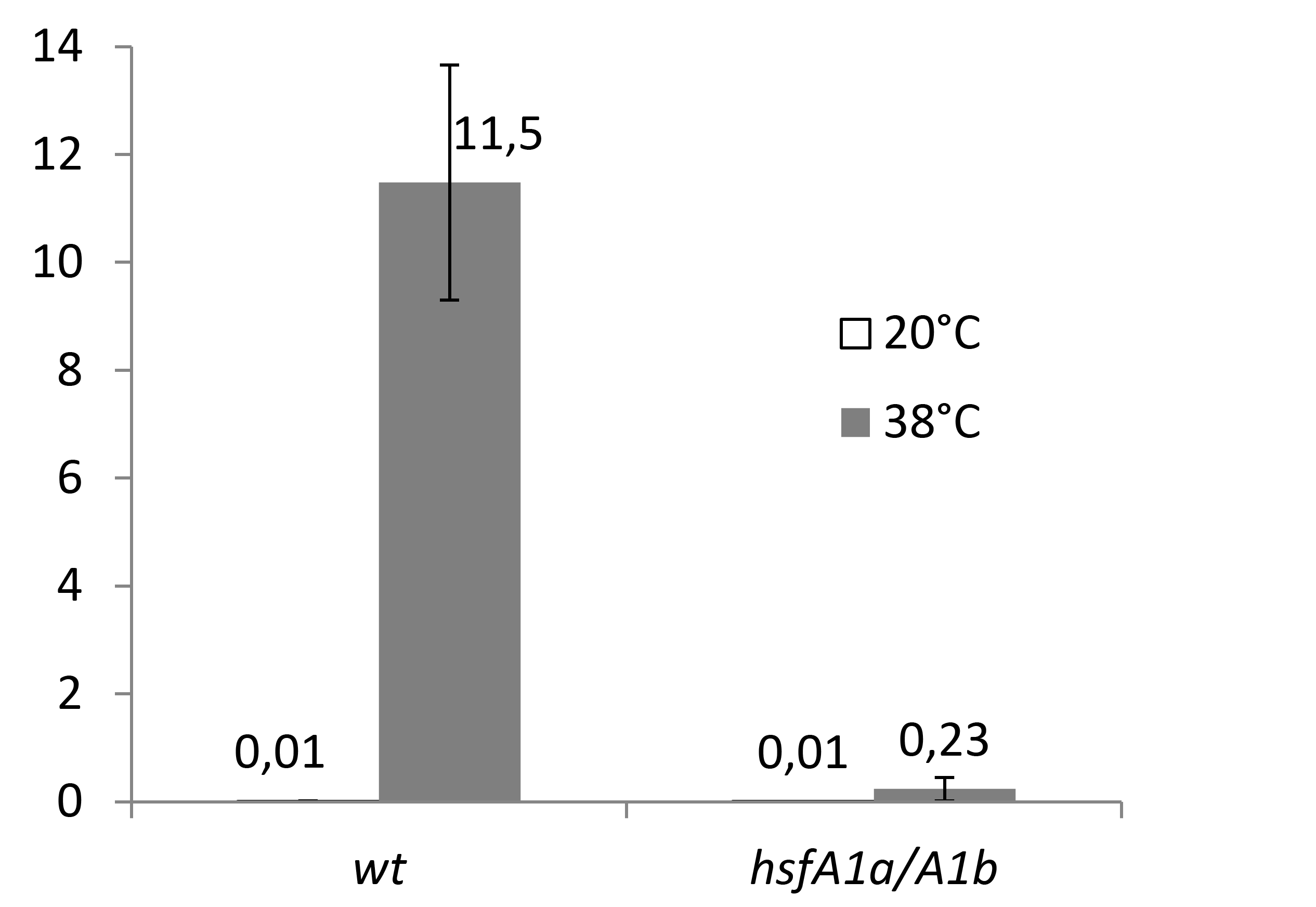

Supplement: Supplementary file 7 — Supplementary material Figure S7: Antisense expression in hsfA1a/hsfA1b plants. Levels of mRNA were determined at control temperature (20°C) and after heat shock (38°C) in wt and hsfA1a/hsfA1b plants. Relative qRT-PCR levels were normalized with respect to Actin2 mRNA (= 100 %). (TIFF 4325 kb) [file 11103_2014_202_MOESM7_ESM.tif]

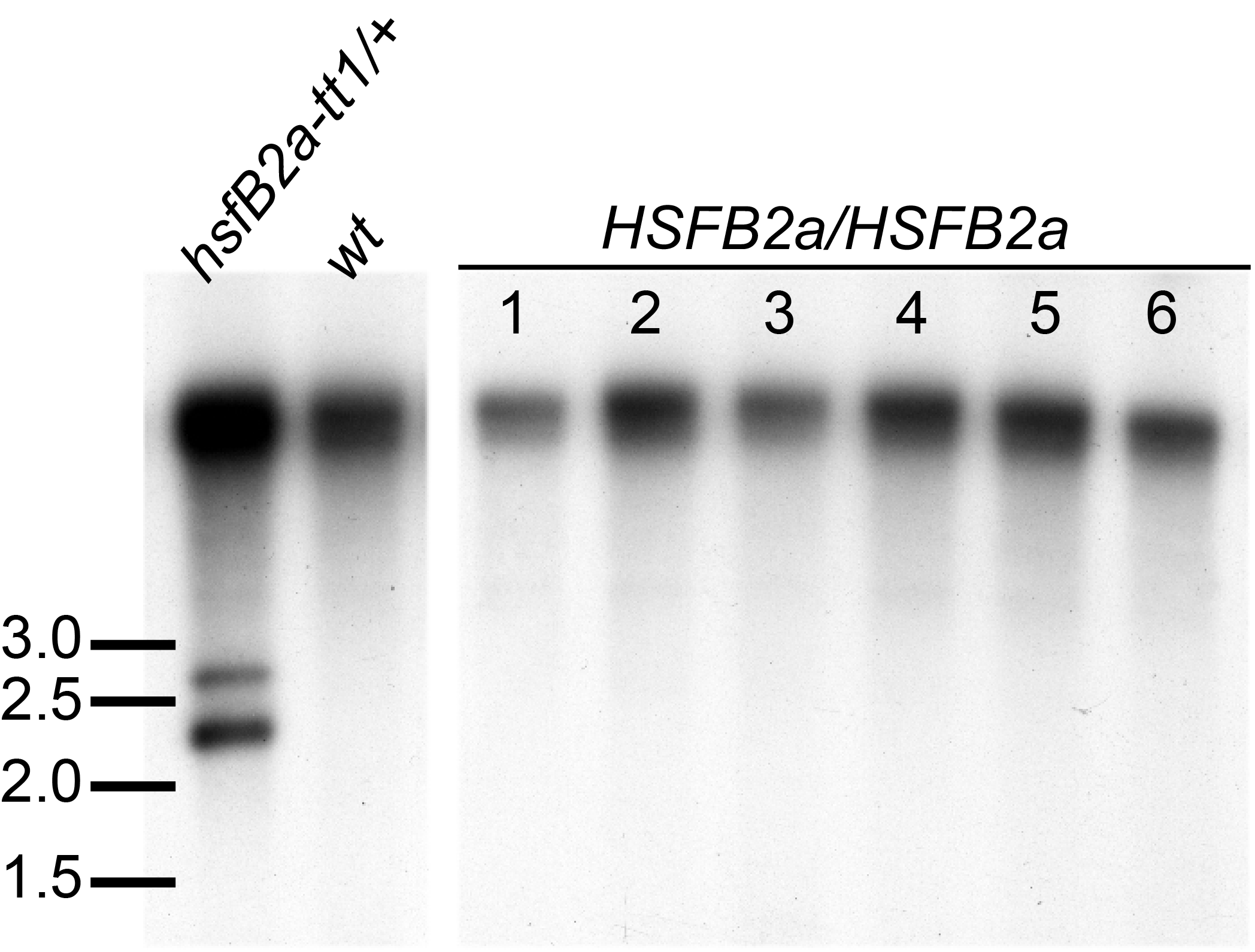

Supplement: Supplementary file 8 — Supplementary material Figure S8: Southern blot hybridization for T-DNA.Genomic DNA of heterozygous mutant (hsfB2a-tt1/+), wild-type plants and the segregating progeny of hsfB2a-tt1/+ with no T-DNA insertion in HSFB2a (HSFB2a/HSFB2a 1-6) was digested with HindIII and the gel blot was probed with a T-DNA specific fragment. Numbers on the left indicate the position of DNA marker bands in kbp. (TIFF 3279 kb) [file 11103_2014_202_MOESM8_ESM.tif]
