# Supplementary material for: Heat shock factor HSFB2a involved in gametophyte development of Arabidopsis thaliana and its expression is controlled by a heat-inducible long non-coding antisense RNA
Source: Plant Mol Biol. 2014 May 30;85(6):541–50. doi: 10.1007/s11103-014-0202-0 (PMC4099531; doi:10.1007/s11103-014-0202-0)
Supplement: Supplementary file 9 — Supplementary material 9 (DOCX 14 kb) [file 11103_2014_202_MOESM9_ESM.docx]

Table S1. Primers used for PCR cloning and PCR analysis

|  |  |
| --- | --- |
| ACTIN2F3 | AAGCTGGGGTTTTATGAATGG |
| ACTIN2R3 | TTGTCACACACAAGTGCATCAT |
| ASB2AF | TTCCTATGGAGTTGCGTGTTT |
| ASB2AR | TGGATGACACATCAAAGCAGA |
| ASFKpnI | GTACGGTACCATATCTACACCACAAATCTAAAC |
| ASRSacI | GTACGAGCTCGTCAAACTTGCTGAGTTTATGGA |
| B2aOXFKpnI | GCGCGGTACCTTACGCTGCTTCTCGATCTTTTA |
| B2aOXRSacI | CGCTGAGCTCTCTACATAATGTCGTTGAAAATCTA |
| B2aPromF | GCTCTAGAAAGTCACGAAAGCAGAAGAAG |
| B2aPromR | GCTCTAGACTTTTGGTTTTGGTCTGGTCTT |
| CaMVKpnR2 | GACCGGTACCCTCTCCAAATGAAATGAACTT |
| CamVSacF2 | TGTGGAGCTCTTTCAGAAAGAATGCTAACCCAC |
| HSF6-ASGWF | GGGGACAAGTTTGTACAAAAAAGCAGGCTTATCTACACCACAAATCTAAACC |
| HSFB2aF2 | gggaaaattccgatgaggag |
| HSFB2aR2 | atcgtctacttcgcgcacata |
| LBa1 | TGGTTCACGTAGTGGGCCATC |
| N512418L | CCACTTGGGGCACACTATATG |
| N512418R | TACCTCCGGGAGAATAACTCC |
| SP6-2B2aR2 | CTAGCTATTTAGGTGACACTATAGAAATCGTCTACTTCGCGCACATA |
| T7-B2aF2 | CTAGCTTAATACGACTCACTATAGGGGGGAAAATTCCGATGAGGAG |
